# Development and validation of a quantitative measure for parent empowerment *via* transformative learning

**DOI:** 10.3389/fpsyg.2022.934142

**Published:** 2022-07-26

**Authors:** Siu-ming To, Lei Yang, Lei Dong, Ming-wai Yan, Yuk-yan So, Mee-yee Chung

**Affiliations:** Department of Social Work, The Chinese University of Hong Kong, Shatin, Hong Kong SAR, China

**Keywords:** parent empowerment, transformative learning, scale development, scale validation, parenting intervention

## Abstract

Although current literature demonstrates how parents benefit from parent empowerment programs, the development of a quantitative measure of parent empowerment has garnered limited attention in parenting research. The goal of this research was therefore to develop and validate a quantitative measure for the assessment of practitioners’ attitudes and competence in parent empowerment. In the process of item generation, the qualitative findings derived from four studies in relation to the perceived outcomes and experiences in parent empowerment were synthesized in the first stage. In the second stage, a list of narratives that articulated different themes of parent empowerment was generated, which resulted in an item pool containing 28 items. In the third stage, the research team converted the 28 items into a survey instrument. In the fourth stage, a first-scale validation study was conducted to explore the factor structure of the initial 28-item questionnaire. The exploratory factor analysis on the first sample of 366 practitioners yielded a twofold factor structure with 17 items, including practitioners’ attitudes in parent empowerment and practitioners’ competence in parent empowerment. In the final stage, a second-scale validation study was undertaken to verify the fit of the twofold factor structure. A confirmatory factor analysis on the second sample of 170 practitioners demonstrated a good model fit. The results of reliability tests for the whole scale and two subscales also indicate satisfactory internal consistency. The Parent Empowerment *via* Transformative Learning Questionnaire (PETLQ) was thus developed and confirmed as a scale with sufficient factorial validity and internal consistency to be used for assessing parenting practitioners’ attitudes and competence in parent empowerment and for evaluating the effectiveness of parent empowerment programs.

## Introduction

The current social concerns over parenting in many societies have led to much intellectual discussion about the purpose and direction of parenting intervention (e.g., Lam et al., 2019; To et al., 2019b). Social discourse surrounding globalization has convinced many parents that their children will experience massive economic, social, and technological changes in the near future, leaving many parents scrambling to find ways to help their children grow, adapt, and survive amidst the changing global landscape. In addition, many parents are now seeking professional advice or services because of the strong impression that such professionals are experts on child development and can therefore guide and instruct parents on what to do (Leung and Lam, 2009; Lam and Kwong, 2012). Such prevailing beliefs may have contributed heavily to an emerging set of standards placed upon today’s parents, who face increasing pressures to adhere to such expectations and to partake in parent education programs that will ultimately enhance their children’s “success” later on. In view of such a changing ecology of parenting, parents have been found to experience a sense of powerlessness, generalized distrust, and alienation from resources for social influence (Lam and Kwong, 2012; To and Chan, 2013; Lam et al., 2018; To et al., 2018b).

This feeling of powerlessness, self-blame, and self-doubt among parents suggests the need for a new paradigm in parent education. Alternative approaches can benefit from paying attention to how social and cultural contexts influence parents’ beliefs, strengthening their personal growth, promoting critical dialog, and enhancing mutual support among parents. In recent years, empowerment has become an attractive concept in the development of parenting intervention and parent education services. According to Lam (2003), the goal of parent empowerment is to activate the strengths, competence, and possibilities for change that exist in parents and in the social context. Instead of transmitting knowledge and skills regarding parenting, practitioners who emphasize parent empowerment tend to embrace parents’ life experiences and facilitate critical reflection about these experiences. Empowerment-oriented practitioners also help parents develop their own beliefs and perspectives in a critical and reflexive way, which can then guide their day-to-day parenting practices. Consequently, parent empowerment has tremendous potential to address the problems inherent in expert-led and deficit-based parenting interventions while shedding light on how to develop more parent-focused, strength-based, and integrated practices.

While current literature demonstrates how parents benefit from parent empowerment programs (e.g., Nieves et al., 2021), few studies elaborate on the perceptions and abilities of practitioners who facilitate the collaborative learning journey. In fact, previous research on community empowerment interventions has indicated that practitioners, as co-learners during the empowerment process, gradually adopt a more situated learning perspective by highlighting flexibility, support, and holism (Quillinan et al., 2019). However, the development of a quantitative measure of parent empowerment has garnered limited attention in parenting intervention research. Most of the relevant studies on parent empowerment have used qualitative methods, such as individual interviews and focus groups, to understand how participants individually and collectively make sense of their experiences in various programs.

That said, a few quantitative studies have been undertaken to examine the concept of parent empowerment or the outcomes of parent empowerment programs. For instance, a study undertaken by Rodriguez et al. (2011) found that the Parent Engagement and Empowerment Program, which aims to improve children’s mental health, helped increase family empowerment, mental health services efficacy, and self-assessment of skills among participants. This study adopted the Family Empowerment Scale (FES) that was originally used to assess empowerment in families whose children have emotional disabilities (Koren et al., 1992). Moreover, Freiberg et al. (2014) constructed the Parent Empowerment and Efficacy Measure (PEEM), which aims to enhance the accountability and effectiveness of family support services by measuring participants’ sense of control or capacity to meet the challenges in parenting. Recently, based on a sample of parents from low-income families, Figueroa et al. (2020) developed a self-administered questionnaire on parental health-related empowerment. Nevertheless, there are still very few quantitative measures targeting the construct of parent empowerment in the area of parenting intervention and parent education. There is also a scarcity of measures adopting a “bottom-up” approach to scale development (Hinkin, 1998), which can be understood as using participants’ direct experiences in parent empowerment programs to generate items of a related scale. Despite the increasing use of parent empowerment as a concept to guide the design of parenting practice, there is thus a pressing need to develop psychometrically valid and reliable tools for measuring the unique features of parent empowerment, especially from the perspective of practitioners who may both enable and constrain the actualization of parent empowerment (Lam and Kwong, 2012).

## Parent empowerment informed by transformative learning

Considering that parent education programs emphasizing the transmission of knowledge and skills might remind parents of their deficits in parenting (To et al., 2013), a transformative learning perspective, which is a well-established concept in adult education, can offer a theoretical framework for exploring the components and content areas of parent empowerment. Whereas parent education often adopts a transmission perspective, which assumes learners are passive and will look to the educator to pass down relevant information, rules, and values (Pope and Denicolo, 2001), a transformative learning approach generally posits learners as active participants in the learning process. Mezirow (2000) defines transformative learning as “the process by which we transform our taken-for-granted frames of reference to make them more inclusive, discriminating, open, emotionally capable of change, and reflective so that they may generate beliefs and opinions that will prove more true or justified to guide actions” (p. 7). As a result of such transformation, individuals develop a more dependable frame of reference and gain greater control over their lives, hence becoming socially responsible decision makers who actively negotiate for and act upon their goals, values, feelings, and meanings rather than being subjected to the discretion of others or to the situation at hand. In sum, transformative learning aims to help individuals develop autonomy and make informed decisions. Furthermore, this approach places focus on the learner as a unique individual and on the surrounding social variables and implications.

Empowerment and transformative learning coalesce around the facilitation of individuals as well as their interactions and relationships with others and the social world in effecting personal and social change (Sokol and Cranton, 1998). Programs and services adopting both approaches provide opportunities for individuals to critically reflect on their values and perspectives, thereby becoming autonomous, socially responsible, and informed decision makers who can forge their own viewpoints and actions without any oppressive constraints. In addition, practitioners who are familiar with transformative learning and empowerment approaches are more cognizant of the hegemonic nature of the current practices in parent education. Similarly, these practitioners may also be better trained to truly respect and empower parents and to help parents develop a critical awareness and engage in reflexive parenting (Leung and Lam, 2009). Furthermore, both approaches may prove to be highly effective in nurturing collaboration and mutual support among parents. As such, the adoption of a transformative learning perspective can help enrich and deepen our understanding of parent empowerment.

## Components of transformative learning and their relations to parent empowerment

Since it is built upon various theoretical underpinnings such as humanism-existentialism, critical theory, and constructivism, transformative learning holds various assumptions and consists of different aspects stemming from its diverse theoretical origins. Thus, it is very difficult, if not impossible, to develop a single, generic scale to capture every aspect of transformative learning (Romano, 2018). Moreover, the process and outcomes of transformative learning may vary according to context and those involved (Stuckey et al., 2013). Therefore, a more feasible approach would be to develop instruments that are specific to the target and type of change sought (Romano, 2018). In this regard, the following essential components of transformative learning and their relations to parent empowerment are highlighted.

### Centrality of experience

Transformative educators view learners as self-directing individuals who can actively make sense of their lived experiences, derive meaning from information or experiences, and develop their own perspectives and viewpoints (Sokol and Cranton, 1998). The life experiences of individuals therefore have a critical role to play in facilitating learning and critical reflection (Taylor, 2009). Given that experiential learning and life experiences provide “pedagogical entry points” (Lange, 2004), the transformative learning approach assumes that incorporating learners’ lived experiences will offer opportunities for engaging in critical reflections about values, perspectives, and purpose, potentially leading to a transformative experience or a new perspective (Taylor, 2009).

Applying this component of transformative learning in understanding parent empowerment, it is clear that the information provided by parenting experts cannot replace the tacit knowledge generated by parents’ own lived experiences (To et al., 2015). By understanding the importance of their own inner resources and experiences, parents may feel more confident in interacting with their children and participating in their children’s life development without relying extensively on external support (To and Chan, 2013). Therefore, helping parents to review and reflect on their lived experiences is a central part of a transformative learning approach to parent empowerment.

### Holistic orientation

Believing that learning is not confined to the head, transformative learning emphasizes a holistic orientation to education and encourages the engagement of other ways of knowing such as affective and relational (Taylor, 2009). Affective knowing, which involves developing an awareness of emotions, is important for transformative learning (Taylor, 2009). Meanwhile, transformative educators also use different means like music or arts and other expressive ways of knowing to evoke experiences for greater exploration, thus creating a learning environment conducive to holistic development (Taylor, 2009).

A transformative learning approach to parent empowerment aims for a holistic approach to parenting. This includes both the personal growth of parents and a strengthened sense of parental competence (Lam, 2003). It believes that the personal growth and learning involved in parenthood is a lifelong journey, and thus, parent empowerment programs should help parents develop self-awareness and sensitivity toward others in order to be fully immersed in the mindset that parenthood is a challenging yet rewarding journey. Parents should also be helped to access necessary support and to participate in decision-making in various domains and levels of parenthood.

### Contextual understanding of knowledge

Knowledge can be derived from a variety of sources (Griffith and Frieden, 2000), but access to knowledge can often be restricted by social, cultural, or historical networks constituted by the interweaving of power and knowledge (Mezirow, 2000). Therefore, it is important to recognize the influences and assumptions of social, cultural, and historical networks and to critically reflect on how related ideologies may impede autonomous learning (Mezirow, 2000). Based on the assumption that knowledge needs to be understood in relation to the surrounding context (Griffith and Frieden, 2000), transformative learning programs or strategies rely heavily on context and its implications for learners (Taylor, 2009). In this regard, context comprises the immediate learning environment, the personal circumstances of learners, and any contexts that have shaped society (Taylor, 2009).

Parent empowerment programs using this approach see the importance of using transformative learning to explore how domination and oppression are maintained through taken-for-granted assumptions, hoping to encourage alternative readings of experience free from domination and oppression. Such an approach provides opportunities for parents to critically reflect on their values and perspectives, thereby becoming autonomous, socially responsible, and informed decision makers in childrearing (Stuckey et al., 2013). Other positive consequences of this approach include stronger interpersonal relationships and positive social change.

### Communicative learning

The use of dialog is a means through which critical reflections can further one’s transformation with the self or with others (Taylor, 2009). Dialog used in transformative learning comprises highly personal, self-disclosing conversations that demonstrate a trust between participants, who are trying to reach an agreement, embrace differences, explore other points of views, and consider reframes in their own thinking (Mezirow, 2000; Traverso-Yépez, 2008). Conditions that create an environment for reflective dialog to occur include freedom from coercion and distorting self-deception, an openness to alternative points of view, empathy and concern about how others think and feel, and an equal opportunity to participate (Mezirow, 2000).

There are two aspects of communicative learning in parent empowerment programs. First, a transformative learning approach promotes the importance of a strong parent–child relationship and the opportunities for parent–child dialog. Specifically, cultivating a deep, sentimental relationship with the child and being able to identify with the child’s experiences are both necessary components for a strong parent–child relationship. When parents and children are actively involved in genuine dialog and shared activities, a sense of connection can naturally form (To and Chan, 2013). So, rather than using various parenting skills in settling the power and control struggle between the parent and child, parents may find greater joy and fulfillment from their role as a parent when their relationship with the child is premised on constructive and meaningful interactions.

Second, practitioners adopting this approach to parent empowerment strive to cultivate a constructive environment for mutual support and learning among parents. Meaningful conversations and mutual support from peers in parent groups can stimulate parents’ continuous growth and the development of parent empowerment programs in a sustainable way. Support networks and learning communities can also be formed as a result of such parent empowerment initiatives.

The above key components suggest that parent empowerment can be generated in a number of ways through the lens of transformative learning. The synthesis of these components opens up the possibility for developing a tentative list of dimensions and expected outcomes of a transformative learning approach to parent empowerment. A rigorous psychometric approach can thus be adopted to generate items and validate a quantitative measure that can be used to understand the perspective of practitioners engaging in parent empowerment interventions and to assess possible changes made by practitioners participating in transformative learning-based training programs. Meanwhile, previous literature points out that a transformative learning approach requires practitioners to have a deep understanding not only of their skills and abilities that allow for culturally responsive practices in collaboration with learners, but also of their mindsets and attitudes toward the nature of learning and transformation (Taylor and Cranton, 2012; Quillinan et al., 2019). Thus, it seems warranted to measure both the attitudes and competence of practitioners to capture their perceptions of reflections and practices in the transformative learning process (Baartman and De Bruijn, 2011).

The goal of this research was thus to develop and validate a quantitative measure for the assessment of practitioners’ attitudes and competence in parent empowerment *via* transformative learning. To achieve this goal, we examined the factor structure and psychometric properties of the proposed questionnaire.

## Materials and methods

### Stage 1: Re-analysis and synthesis of narratives of parents’ perceived outcomes and experiences in parenting programs adopting a transformative learning approach

To construct and validate a quantitative measure for parent empowerment informed by transformative learning, we first synthesized the qualitative findings derived from four studies in relation to the perceived outcomes and experiences in parenting programs adopting a transformative learning approach. All parenting programs reported in these studies were undertaken by members of the Hong Kong Parent Education Association, a non-profit organization formed by a group of social workers and parent education practitioners. The practitioners of these programs were equipped themselves with rich knowledge and experience in adopting a transformative learning approach to designing and implementing parent education programs (To et al., 2013). Therefore, the themes elicited from the narratives of the participants can reflect not only their perceptions of the programs, but also their perceptions of a transformative learning approach to parent education as a whole (To et al., 2013).

In Study 1 (To et al., 2013), a total of 17 parents joined three post-intervention focus groups after the program. They were all Hong Kong Chinese parents with at least one child who was receiving education in a local secondary school (equivalent to middle and high school). In Study 2 (To et al., 2014), a total of 20 participants joined three post-intervention focus groups after the program. They were all Hong Kong Chinese parents with at least one child studying in a nursery school. In Study 3 (To et al., 2015), a total of 25 participants joined five post-intervention focus groups after the program. All were Hong Kong Chinese parents who had at least one child in kindergarten, primary school (equivalent to elementary school), or secondary school. In Study 4 (To et al., 2018b), a total of 45 participants joined 11 post-intervention focus groups after the program. All of them were Hong Kong Chinese parents with at least one child studying in nursery school or primary school. In sum, we gathered the narratives of 107 Hong Kong Chinese parents from a total of 22 focus groups regarding their perceived outcomes and experiences in parent education programs informed by transformative learning. All these narratives provided specific information about the themes or content areas related to parent empowerment *via* transformative learning as perceived by local parents.

### Stage 2: Item generation

In the second stage, two of our team’s researchers re-analyzed and synthesized the narratives derived from these focus group studies to generate a list of participants’ narratives that articulated different themes of parent empowerment *via* transformative learning (see Table 1), including (1) emphasis on parents’ own experiential knowledge and meaning-making in parenthood, (2) facilitation of self-integration and self-enrichment through telling life stories, (3) generation of critical reflections on the dominant discourses and ideologies in parenting, (4) cultivation of parent–child connectedness and improvement in parent–child relationships, (5) understanding of children’s developmental needs, emotions, potentials, and individuality, and (6) cultivation of mutual support and mutual learning through small group sharing. The articulation of these themes was guided by the principle that they were elicited from at least two focus group studies and that they could reflect the general perceptions of the participants regarding their learning outcomes and experiences. The research team also identified 12 sub-themes under the six aforementioned themes: (1) parents openly and honestly reflect on their everyday parental experiences, (2) parents explore and deepen the meaning of being a parent, (3) parents organize their life stories and growth experiences, (4) parents experience personal growth, (5) parents have deep critical reflections on the sociocultural context in which they are situated, (6) parents discover their own resources and abilities to face the challenges of being a parent, (7) parents place more emphasis on the relational connection with their children, (8) parents use different ways to deepen their relational connection with their children, (9) parents understand their own developmental and emotional needs so that they can better understand their children’s developmental and emotional needs, (10) parents explore their own way to raise their children, (11) parents trust and support each other, and (12) parents build a community to mutually support and learn from each other.

**TABLE 1:** Themes and examples of narratives of parent empowerment *via* transformative learning.

Dimension	Theme	Example of narratives
(A) Emphasis on parents’ own experiential knowledge and meaning-making in parenthood.	(1) Parents openly and honestly reflect on their everyday parental experiences. (2) Parents explore and deepen the meaning of being a parent.	“Although we had similar experiences in parenting, some group members had different reflections on those experiences. Their reflections stimulated me to use other perspectives to think about the meaning of parenthood.” (as cited in To et al., 2013, p. 86). “At this moment of life, this person [the child] is the most important person to me. You will refocus your life according to this understanding. This helps you filter out a lot of things in life and then you can restart your life journey.” (as cited in To et al., 2018b, p. 175).
(B) Facilitation of self-integration and self-enrichment through telling life stories.	(3) Parents organize their life stories and growth experiences. (4) Parents experience personal growth.	“You can remember the beautiful life episodes in this process, which can help reassert a sense of mastery in facing future challenges. You will not only focus on problems or family conflicts, which are in fact trivial. There were many good things that you did in the past such as working together with your spouse to build the family and nurture your children. When you think about these, you will experience personal growth and development.” (as cited in To et al., 2013, p. 87). “The program gave me an opportunity of self-evaluation, and I could reorganize my life experiences, no matter positive or negative. It was good for me because all these experiences have affected my life attitude. After the reorganization of my life experiences, I found that life should be very simple in the way that we should cherish our children.” (as cited in To et al., 2015, p. 107).
(C) Generation of critical reflections on the dominant discourses and ideologies in parenting.	(5) Parents have deep critical reflections on the sociocultural context in which they are situated. (6) Parents discover their own resources and abilities to face the challenges of being a parent.	“I have received many messages from society regarding the roles of being a parent. The practitioner helped us challenge the old way of thinking. [He] did not talk much about theories. He used many daily life examples and his own life experiences to help us reflect.” (as cited in To et al., 2013, p. 86). “As a person, I used to be very doubtful about myself. But after the workshop, I became more confident. Now I just do what I think is right.” (as cited in To et al., 2018b, p. 176).
(D) Cultivation of parent–child connectedness and improvement in parent–child relationships.	(7) Parents place more emphasis on the relational connection with their children. (8) Parents use different ways to deepen their relational connection with their children.	“My child is my “flesh and bones.” It’s not a responsibility but a life devotion to take care of my children. It’s natural for you to take care of your leg when it hurts because it is a part of your body. I have a stronger sense of the parent–child connection after participating in this group.” (as cited in To et al., 2014, p. 52). “I came home and looked at my daughter — she is really my “flesh and bone.” The practitioner encouraged us to review the photos that were taken when my child was born. At that time, I often asked myself how I could take care of my baby. Now I am amazed by my ability to bring her up.” (as cited in To et al., 2015, p. 108).
(E) Understanding of children’s developmental needs, emotions, potential, and individuality.	(9) Parents understand their own developmental and emotional needs so that they can better understand their children’s developmental and emotional needs. (10) Parents explore their own way to raise their children.	“These three workshops can provide opportunities for me to think about my life, to reorganize, and to address issues. When anger emerges, I will be alert and remind myself that this has nothing to do with my child’s behavior. Then, I can calm down.” (as cited in To et al., 2018b, p. 177). “I could manage my own life when I grew up. Why can’t my child? Now I always remind myself that I should allow more space and freedom for him to grow. I feel more relaxed now. I try my best to help my child, but I resist putting so much pressure on myself. It seems that everything has become smoother!” (as cited in To et al., 2015, p. 108).
(F) Cultivation of mutual support and mutual learning through small group sharing.	(11) Parents trust and support each other. (12) Parents build a community to mutually support and learn from each other.	“We joined our hands to go through the process of life integration. We shared our life experiences with each other. We also talked about our experiences in parenting. I could learn from my group members’ experiences and know how to preserve a positive attitude to face the difficulties.” (as cited in To et al., 2014, p. 53). “When I listened to other group members’ sharing, I could learn about how other parents coped with problems in childrearing. I could take their experience and wisdom as my reference. Moreover, through the sharing of male group members, I could have a deeper understanding of males’ perceptions and viewpoints. Therefore, I knew how to put myself in my family members’ shoes.” (as cited in To et al., 2015, p. 109).

Then, the other three team members discussed these preliminary themes and found that when applying these themes in studying the perceptions of practitioners with regard to parent empowerment *via* transformative learning, these themes could be re-categorized into two major components, namely attitudes (i.e., practitioners’ beliefs and motivation in adopting a transformative learning approach to parent empowerment) and competence (i.e., practitioners’ sense of competence in adopting a transformative learning approach to parent empowerment) (Baartman and De Bruijn, 2011). Based on these six themes, 12 sub-themes, and two major components (i.e., attitudes and competence), these three project-team researchers independently generated different items of the instrument. Then, they cross-evaluated the items generated by each researcher, and the items receiving unanimous agreement were retained in the item pool. At the end of this process, the resulting item pool contained 28 items regarding the outcomes and experiences of parent empowerment. During the process of item generation, the researchers were careful to keep items concise and focused, and avoid double-barreled questions and complicated syntax to decrease item ambiguity (Podsakoff et al., 2003).

### Stage 3: Pilot survey

In the third stage, the research team converted the 28 items into a survey instrument. Each item was rated along a seven-point Likert scale. This preliminary survey instrument was pilot-tested with a non-random sample of 51 practitioners who had rich professional knowledge and experience in adopting a transformative learning approach to parent education. Besides filling out the questionnaire, they were asked to give comments on the questionnaire items. Among the pilot study respondents, 9.8% were male and 90.2% were female. Of the 51 respondents, 2.2% were aged 21–30, 15.7% were 31–40, 43.1% were 41–50, 35.1% were 51–60, and 3.9% were 61 or above. In terms of education level, 3.9% had college-level education or below, 19.6% university level, 74.5% master’s level, and 2.0% doctoral level. Then, the research team retained or modified the items of the questionnaire based on the results of the preliminary analysis, including the initial reliability analysis and item analysis, as well as the practitioners’ written comments. They thus developed a 28-item questionnaire and then tested it in the first validation study.

### Stage 4: First validation study

In the fourth stage, we conducted the first-scale validation study to explore the factor structure of the initial 28-item questionnaire on data collected from the pre-test assessment of a parent education project in Hong Kong. In this stage, we sent invitation letters to ten collaborating social service agencies to solicit their support in recruiting practitioners to participate in this study, and a total of 366 practitioners were surveyed in the first-scale validation study. Exploratory factor analysis (EFA) was performed to examine the underlying factor structure of the 28-item questionnaire.

### Stage 5: Second validation study

In the final stage, we conducted the second-scale validation study to verify the fit of the factor structure derived from the EFA of the first study. Following Hinkin’s (1998) recommendation on the steps of scale development and validation, we performed a confirmatory factor analysis (CFA) on data collected from a new sample, which was taken from the intermediate-test assessment of this parent education project. After excluding the practitioners who had been surveyed in the first validation study, a total of 170 practitioners participated in the second validation study.

Prior to conducting this research, we obtained ethics approval from the Survey and Behavioral Research Ethics Committee of the affiliated institution. The team members obtained informed written consent from the practitioners prior to their participation, and the consent form clearly demonstrated the research objective and the way that the data would be processed. It also emphasized that their participation was completely voluntary and anonymous and that their information would be kept strictly confidential (Podsakoff et al., 2003).

## Measures

### Parent empowerment *via* transformative learning questionnaire

As demonstrated, five stages were completed to develop the PETLQ. The initial PETLQ included 28 items, each rated on a seven-point Likert scale. As proposed and hypothesized, the PETLQ was made up of the attitude subscale (14 items) and the competence subscale (14 items). A sample item from the attitude subscale includes “I think parent work should involve helping parents critically reflect on various prevailing childrearing practices or discourses in society.” A sample item from the competence subscale includes “I am able to help parents organize their life stories and growth experiences.” For each subscale, the scores of the items are summed as the subscale score. A higher subscale score reflects a higher degree of agreement with the attitudes or competence in adopting a transformative learning approach in parent empowerment. The original version of the questionnaire was in Chinese as the items were derived from interview texts with the original linguistic expressions retained as faithfully as possible. Given the need to disseminate research-based knowledge in research papers, the original Chinese version of the PETLQ was translated into English and then back-translated into Chinese. With several modifications and wording revisions based on the results of translation and back-translation, the items of the PETLQ were finalized. A copy of the questionnaire is available from the first author upon request.

### Sociodemographic characteristics

Age, gender, educational level, and relevant information about work experience were collected. Descriptive analyses were performed to obtain the frequencies and percentages or mean and standard deviations of demographic variables. The details of the sociodemographic characteristics are presented in Table 2.

**TABLE 2:** Sociodemographic characteristics of participants.

Variable	*n* = 366 (%)^a^	*n* = 170 (%)^b^
**Age group**		
20–30	93 (25.6)	46 (27.1)
31–40	171 (47.1)	66 (38.8)
41–50	71 (19.6)	39 (22.9)
51 or above	28 (7.7)	19 (11.2)
Missing	3	0
**Gender**		
Male	76 (20.8)	41 (24.1)
Female	289 (79.2)	129 (75.9)
Missing	1	0
**Having child(ren) or not**		
Yes	133 (36.4)	67 (39.4)
No	232 (63.6)	103 (60.6)
Missing	1	0
**Education level**		
College or below	54 (14.8)	35 (20.6)
University	169 (46.3)	78 (45.9)
Postgraduate or above	142 (38.9)	57 (33.5)
Missing	1	0
**Years of experience in the current job position**		
1 year or below	80 (22.7)	16 (9.4)
1 year above to 5 years	118 (33.4)	79 (46.5)
5 years above to 10 years	68 (19.3)	30 (17.6)
10 years above to 15 years	37 (10.5)	19 (11.2)
15 years above to 20 years	20 (5.7)	13 (7.6)
20 years above	30 (8.5)	13 (7.6)
Missing	13	0
**Frequency of participation in parent education training in the last year**		
None	91 (25.0)	41 (24.1)
1–3 times	208 (57.1)	104 (61.2)
4–6 times	44 (12.1)	20 (11.8)
7–9 times	9 (2.5)	4 (2.4)
10 times or above	12 (3.3)	1 (0.6)
Missing	2	0

^a^Sample size of the EFA; ^b^sample size of the CFA.

### Data analysis

All data analyses were conducted using SPSS 28 and Amos 25. In the first-scale validation study, we conducted an EFA to identify the factor structure for the items of the PETLQ. After the factor structure had been explored, reliability analysis and item analysis were carried out based on this sample. In the second-scale validation study, we conducted a CFA to test whether the data fit the hypothesized factor structure. We assessed the goodness-of-fit using a variety of fit indices. A relative chi-square value (CMIN/df) less than 5 (Schumacker and Lomax, 2004), a root-mean-square error of approximation (RMSEA) value lower than 0.08 (Browne and Cudeck, 1993), an incremental fit index (IFI) and a Tucker–Lewis index (TLI) score higher than 0.90 (Bollen, 1989), and a comparative fit index (CFI) value higher than 0.93 (Byrne, 1994) were set as the criteria for model acceptability.

## Results

In the first-scale validation study, an EFA was performed to examine the underlying factor structure of the 28-item questionnaire based on a sample of 366 participants. We used principal axis factoring (PAF) with an oblique rotation to produce five factors with eigenvalues > 1.0. However, eight items (items 2, 4, 8, 11, 16, 22, 25, and 27) were deleted because of their scattering in three different factors with weak loadings. Then, we conducted PAF with an oblique rotation on the remaining 20 items again, which yielded three factors with eigenvalues exceeding unity. Due to weak loadings below 0.40, two items (items 12 and 19) were deleted (Tabachnick and Fidell, 2007). Meanwhile, we also evaluated the cross-loadings of a variable by the ratio of their squared loadings. As suggested by Hair et al. (2019), both problematic and potential cross-loadings (i.e., ratio between 1.0 and 2.0) can be deleted. Thus, item 7 was deleted in this step. After that, further analysis with an oblique rotation was performed on the remaining 17 items. The Kaiser–Meyer–Olkin measure of sampling adequacy and the Bartlett’s test of sphericity were explored to assess the appropriateness of factor analysis (Hair et al., 2019). At this stage, the Kaiser–Meyer–Olkin value was 0.91, and Bartlett’s test of sphericity reached statistical significance (*p* < 0.001), indicating that the sample met the criteria for factor analysis (Hair et al., 2019).

As shown in Table 3, according to the extraction of factors with eigenvalues > 1.0, a twofold factor structure was generated. These factors explained 51.4% of the total variance. Factor 1 accounted for 36% of the total variance and contained nine items; factor 2 accounted for 15.4% of the total variance and contained eight items. All items had single dominant factor loadings higher than 0.4. Factor 1 (nine items) measured competence, and Factor 2 (eight items) measured attitudes. The communalities of most variables were higher than 0.4, with a mean level of 0.5, indicating that the reliability of the indicators is acceptable (Costello and Osborne, 2005; Hair et al., 2019).

**TABLE 3:** Exploratory factor analysis and item analysis of the 17-item parent empowerment *via* transformative learning questionnaire (PETLQ).

		Item analysis (*n* = 366)			Factor loadings from EFA (n = 366)		
			
		*M*	*SD*	Item-total^	Com^	1	2
**Factor 1: Competence (variance explained: 36.0%)**							
18.	I have the ability to facilitate parents’ personal growth.	4.967	0.998	0.790	0.689	0.825	0.014
23.	I am able to assist parents to understand their own developmental and emotional needs so that they can better understand their children’s developmental and emotional needs.	5.157	0.888	0.753	0.638	**0.822**	−0.082
24.	I can help parents explore their own way to raise their children.	5.111	0.840	0.705	0.554	**0.762**	−0.060
17.	I am able to help parents organize their life stories and growth experiences.	4.828	0.984	0.725	0.575	**0.753**	0.016
20.	I am able to help parents discover their own resources and abilities to face the challenges of being a parent.	5.219	0.848	0.718	0.568	**0.731**	0.061
28.	I am able to assist parents to explore and deepen the meaning of being a parent.	4.818	0.974	0.723	0.575	**0.724**	0.090
21.	I am able to help parents deepen their understanding of the relational connection with their children.	4.816	1.032	0.688	0.519	**0.716**	0.014
26.	I have confidence in helping parents build a community to mutually support and learn from each other.	5.028	0.976	0.627	0.440	**0.688**	−0.089
15.	I am able to assist parents to reflect on their everyday parental experiences openly and honestly.	5.230	0.911	0.702	0.565	**0.674**	0.178
**Factor 2: Attitudes (variance explained: 15.4%)**							
14.	I think it is more important for parents to explore and deepen the meaning of being a parent than to learn correct parenting knowledge and skills.	5.470	1.099	0.608	0.505	−0.067	**0.730**
9.	I think it is more important for parents to experience deep relational connection with their children than to learn communication methods and skills.	5.050	1.340	0.564	0.440	0.084	**0.630**
10.	I think that when parents can understand their own developmental and emotional needs, they can better understand their children’s developmental and emotional needs.	5.915	0.934	0.527	0.365	0.006	**0.602**
5.	To facilitate parents’ personal growth and integration of lived experiences, I think it is necessary to assist parents in narrating and reflecting on their life stories.	5.764	0.903	0.459	0.271	−0.064	**0.538**
13.	I think it is more important to help parents build a community to mutually support and learn from each other than to find professionals to teach parents.	5.626	0.968	0.431	0.255	0.012	**0.501**
1.	I think that whether parents can openly and honestly reflect on their everyday parental experiences is more important than whether they can acquire correct childrearing knowledge and skills.	5.655	1.066	0.441	0.255	0.016	**0.499**
3.	I think the primary goal of parent work should be to facilitate parents’ personal growth and their integration of lived experiences.	5.758	0.858	0.439	0.279	0.125	**0.473**
6.	I think parent work should involve helping parents critically reflect on various prevailing childrearing practices or discourses in society.	4.728	1.376	0.374	0.178	−0.020	**0.428**
**Total variance explained: 51.4%**							
McDonald’s omega (*n* = 366):0.857						0.919	0.775

Item-total^, item-total correlation; Com^, communalities. The bold values reflect the twofold factor structure of this measure.

Item analyses, including the item-total correlations and reliability tests, are also demonstrated in Table 3. The McDonald Omega coefficient (ω) for the whole scale was 0.857. The subscale Omega coefficients were 0.775 (PETLQ-attitude subscale) and 0.919 (PETLQ-competence subscale). These results indicate satisfactory internal consistency (Green and Yang, 2015; Flora, 2020). Furthermore, the means, standard deviations, and correlations of the two factors and the whole scale are presented in Table 4. The results indicate that these two factors represent separate but related constructs.

**TABLE 4:** Means, standard deviations, and correlations among the subscales and whole scale (*n* = 366).

		1	2	Mean	SD
(1)	Attitudes in parent empowerment *via* transformative learning			5.491	0.667
(2)	Competence in parent empowerment *via* transformative learning	0.316***		5.019	0.733
(3)	Whole scale: parent empowerment *via* transformative learning	0.765***	0.853***	5.241	0.571

***p < 0.001.

**FIGURE 1:**
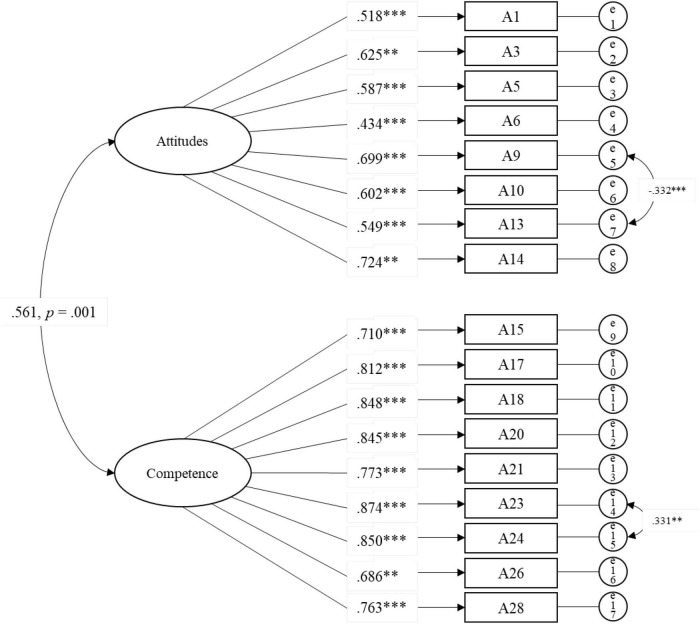
Results of confirmatory factor analysis (*n* = 170). ***p ≤ 0.001, **p ≤ 0.01.

To verify the fit of the twofold factor structure derived from EFA, we conducted a CFA based on a sample of 170 participants. The standardized parameters, path diagrams, and factor loadings are presented in Figure 1. All of the parameter estimates were significant at a level of *p* ≤ 0.001 or *p* ≤ 0.01, and all factor loadings exceeded 0.5 except for that of Item A6 (0.434). Based on the factor loadings, the composite reliability of attitudes and competence was 0.976 and 0.993, respectively, which indicated that all the items consistently measure their corresponding construct (Nunnally and Bernstein, 1994). Meanwhile, covariances were added within the factor because of the high modification index value, which may be caused by the similarity in the wordings and theoretical correlations of these items (Datu and Yuen, 2021). Furthermore, as shown in Table 5, the satisfactory model fit confirmed the structural validity of the scale (CMIN/df = 1.826, RMSEA = 0.070). Likewise, the incremental fit index (IFI = 0.940), the Tucker–Lewis index (TLI = 0.929), and the comparative fit index (CFI = 0.940) also supported a satisfactory model fit. Moreover, the factor correlation between attitudes and competence in parent empowerment *via* a transformative learning approach was significantly correlated in this study (*r* = 0.561, *p* ≤ 0.001).

**TABLE 5:** Goodness-of-fit measures of the parent empowerment *via* transformative learning questionnaire (PETLQ) (*n* = 170).

Model	χ^2^	*df*	CMIN/*df*	RMSEA	IFI	TLI	CFI
Two-factor Structure	211	116	1.826	0.070	0.940	0.929	0.940

## Discussion

This study represents one of the few efforts to develop and validate a quantitative measure for assessing parent education practitioners’ attitudes and competence in parent empowerment informed by transformative learning. Based on the psychological constructs generated from transformative learning-related theories and practices, the PETLQ was confirmed as a scale with sufficient factorial validity and internal consistency to be used for assessing and improving parent empowerment programs.

To start with, although practitioners may have different understandings of the goals and meanings of parent education, the relatively high mean scores and the confirmation of the PETLQ’s two-factor model indicate that participants in general support the development of a transformative learning approach to parent empowerment that pays attention to the lived experiences of parents, the influence of social and cultural contexts in parenting, and the importance of mutual support and learning among parents. One possible explanation is that empowerment, characterized by a personally meaningful, goal-oriented process of increasing power in cognitive, emotional, and interpersonal domains (Cattaneo and Chapman, 2010), is widely accepted by practitioners who want to improve their parenting intervention effectiveness (Rodriguez et al., 2011; Figueroa et al., 2020). Thus, even for practitioners who are not familiar with the concept of transformative learning, they may still agree with some of the core ideas that align with empowerment.

Meanwhile, by comparing the PETLQ’s items with other relevant questionnaires such as Family Empowerment Scale (FES) (Rodriguez et al., 2011), Parental Health-Related Empowerment Scale (Figueroa et al., 2020), or the Parent Empowerment and Efficacy Measure (PEEM) (Freiberg et al., 2014), the PETLQ demonstrates uniqueness since it was constructed based on a dialog between theories and items generated through a “bottom-up” approach (Hinkin, 1998). By integrating participants’ understandings of their experiences into the development of the scale, the PETLQ may better reflect the conceptualization of a transformative learning approach to parent empowerment, which highlights the centrality of experience and a contextualized understanding of knowledge (Taylor, 2009). Moreover, while other scales mainly target parents in special situations (e.g., parents with children with disabilities and health needs), our scale demonstrates wider applicability by targeting practitioners who provide parenting services for different groups of parents.

As for the details in scale validation, based on the results of EFA, four proposed negatively worded items (i.e., item 2: “I think it is necessary to develop a comprehensive parent education curriculum by professionals to enhance parental competence in parenthood”; item 4: “I think the primary goal of parent work should be to teach parents how to nurture their children”; item 8: “I think most parents need to receive education to learn the knowledge and skills in improving parent–child relationships”; and item 11: “I believe parents can raise their children by a certain parent education method that is proven to be empirically effective”) were deleted because they cannot be loaded into the attitude subscale. However, the four corresponding positively worded items (i.e., item 1: “I think whether parents can openly and honestly reflect on their everyday parental experiences is more important than whether they can acquire correct childrearing knowledge and skills”; item 3: “I think the primary goal of parent work should be to facilitate parents’ personal growth and their integration of lived experiences”; item 5: “To facilitate parents’ personal growth and integration of lived experiences, I think it is necessary to assist parents in narrating and reflecting on their life stories”; and item 14: “I think it is more important for parents to explore and deepen the meaning of being a parent than to learn correct parenting knowledge and skills”), which indicate a positive attitude toward a transformative learning approach to parent education, demonstrate sufficient factor loadings onto the attitude subscale. One possible explanation is that those proposed negatively worded items tend to form a different dimension (Merritt, 2012), rather than the opposite end of those positively worded items, which makes them not significantly associated with the factor indicating a positive attitude toward parent education *via* a transformative learning approach. In other words, there may exist different dimensions of parent education (e.g., a transformative and a transmission approach) among some practitioners. For example, while practitioners hold positive attitudes toward parent empowerment in general situations, some of them may consider the special situations faced by different groups of parents (such as parents of children with special educational needs, parents with substance abuse concerns) in which transmission of knowledge and skills in childrearing is essential. Previous literature on the historical changes of education approaches also supports this explanation by arguing that today’s education practices are layered, including didactic, authentic, and transformative approaches (Kalantzis and Cope, 2020).

Another notable finding related to the competence subscale during EFA is that four proposed negatively worded items (i.e., item 16: “I am worried that I cannot understand parents’ distress and concerns”; item 22: “I have no confidence that I can help parents focus on deepening their relational connection with their children rather than solely learning the correct communication methods and skills”; item 25: “I do not know how to facilitate mutual trust and mutual aid among parents”; and item 27: “I am worried that I cannot understand the unique circumstance that each parent is facing”) cannot be loaded into the competence subscale. As with the discussion on negatively worded items in the attitude subscale above, it is possible that these negatively worded items here also tend to form a different dimension, rather than the opposite end of practitioners’ competence in adopting a transformative learning approach. In other words, practitioners may be somewhat worried about adopting a transformative learning approach and somewhat feel confident in adopting this approach. For example, being practitioners informed by transformative learning, they still face paradoxical situations and uncertainties in engaging parents in the transformative learning process which may lead to their continuous reflection on their own positions and approaches (Lam and Kwong, 2012). This finding echoes previous research on the assessment of transformative learning processes that emphasizes anticipating or experiencing uncertainties (Cox, 2021).

## Limitations

There are three major limitations of this study. First, we only adopted self-reported questionnaires to collect data which may increase the threat of social desirability bias. Second, although two samples were used to validate the PETLQ, the generalizability of the findings should be subject to scrutiny because neither were randomized representative samples. Last, while this study targeted parent education practitioners, our team did not develop and validate relevant empowerment scales targeting parents.

## Implications and future research directions

Despite the limitations, the present findings on the development and validation of the PETLQ still yield valuable implications. First, regarding the deletion of negatively worded items, this study implies that scholars have to take the effects of using negatively or reverse worded items into consideration when designing and validating scales (Zhang et al., 2016). Moreover, practitioners and parents may have different perceptions of the positively and negatively worded items in the scale. Thus, future studies could include both practitioners and parents to produce context-specific scales targeting different groups, which may help to depict a more comprehensive picture of the empowerment outcomes and processes.

There are also many practice-related aspects to be further explored in future. One is that the relatively lower mean score of the competence subscale highlights the importance of capacity building for parent education practitioners. Future trainings or workshops targeting practitioners may consider themes based on specific items of this scale, especially for practitioners’ competence in facilitating parents’ personal growth and helping them to explore the meaning of being a parent. Previous research on parent empowerment practices also emphasizes similar training components for practitioners, such as the capacity for reflective listening, showing empathy toward parents, and encouraging mutual support and learning among parents (Day et al., 2012; Quillinan et al., 2019; To et al., 2019a).

Meanwhile, practitioners themselves could design and implement parent empowerment programs with reference to the themes highlighted by the PETLQ. By integrating theoretical elements of transformative learning with participants’ experience in parent empowerment programs, this scale could inspire practitioners to place greater emphasis on helping parents to increase self-understanding, reconstruct parental identities, and deepen parent–child relational connection (Lange, 2004; To et al., 2018a). For example, practitioners could help parents to reflect on dominant parenting discourses and realize the intrinsic value of their lived experience by creating a conversational space for parents to have genuine and constructive dialog with their children or other parents (Leung and Lam, 2009; Lam and Kwong, 2012; To and Chan, 2013).

Finally, for future research related to program evaluation, as a valid and reliable scale, the PETLQ can also be used to evaluate the effectiveness of parent empowerment informed by transformative learning. Based on a systematic review on empowerment interventions with families, Borges Rodrigues et al. (2021) pointed out that current studies lack details regarding how to operationalize key theoretical constructs of empowerment, noting that few studies present a theoretical application at the evaluation stage. Thus, the PETLQ could be used to address this knowledge gap by enabling researchers and practitioners to conduct post-intervention assessment by measuring empowerment constructs informed by transformative learning. Moreover, the evaluation outcomes could help practitioners to guide decisions about how to improve parent empowerment programs.

## Data availability statement

The datasets generated for this study are not readily available due to its ownership by the Hong Kong Jockey Club Charities Trust. Requests to access the datasets should be directed to S-MT, siumingto@cuhk.edu.hk.

## Ethics statement

The studies involving human participants were reviewed and approved by the Survey and Behavioral Research Ethics Committee of The Chinese University of Hong Kong. The patients/participants provided their written informed consent to participate in this study.

## Author contributions

S-MT: project leader, initiated the project, and active in all phases of the project, including design, data collection, data analysis, and writing. LY: active in data collection, data analysis, and writing. LD: active in writing and editing. M-WY: active in data analysis and writing. Y-YS: active in design. M-YC: active in data collection. All authors contributed to the article and approved the submitted version.
